# Body Composition Is Not Related to Structural or Vascular Brain Changes

**DOI:** 10.3389/fneur.2019.00559

**Published:** 2019-05-28

**Authors:** Pauline H. Croll, Daniel Bos, Mohammad Arfan Ikram, Fernando Rivadeneira, Trudy Voortman, Meike W. Vernooij

**Affiliations:** ^1^Department of Epidemiology, Erasmus University Medical Center, Rotterdam, Netherlands; ^2^Department of Radiology and Nuclear Medicine, Erasmus University Medical Center, Rotterdam, Netherlands; ^3^Department of Internal Medicine, Erasmus University Medical Center, Rotterdam, Netherlands

**Keywords:** body composition, brain volume, white matter integrity, cerebral small vessel disease (CSVD), fat mass index (FMI), fat-free mass index (FFMI)

## Abstract

**Background:** It is known that obesity [measured with body mass index (BMI)] relates to brain structure and markers of cerebral small vessel disease (CSVD). However, BMI may not adequately represent body composition. Furthermore, whether those cross-sectional associations hold longitudinally remains uncertain.

**Methods:** Three thousand six hundred and fourty-eight participants underwent baseline (2006–2014) dual-energy X-ray absorptiometry (DXA)-scan to obtain detailed measures of body composition and a magnetic resonance imaging (MRI) scan to assess brain structure. One thousand eight hundred and fourty-four participants underwent a second MRI-scan at follow-up (2010–2017; median follow-up: 5.5 years). To assess cross-sectional and longitudinal associations (measures of change have been calculated) between body composition [BMI, fat mass index (FMI), fat-free mass index (FFMI)], and brain tissue volume (gray matter, white matter, hippocampus), white matter microstructure [fractional anisotropy (FA), mean diffusivity (MD)], and CSVD markers (white matter hyperintensity volume, lacunes, microbleeds) we used multivariable linear and logistic regression models.

**Results:** A higher BMI and FMI were cross-sectionally associated with smaller white matter volumes (difference in Z-score per SD higher BMI: −0.064 [95% CI: −0.094, −0.035]) and FMI: −0.067 [95% CI: −0.099, −0.034], higher FA and MD. A higher FFMI was associated larger gray matter volume (difference: 0.060 [95% CI: 0.018, 0.101]). There was no statistically significant or clinically relevant association between body composition and brain changes.

**Conclusions:** Body composition, distinguishing between fat mass and fat-free mass, does not directly influence changes in brain tissue volume, white matter integrity and markers of CSVD. Cross-sectional associations between body composition and brain tissue volume likely reflect cumulative risk or shared etiology.

## Introduction

The increasing prevalence of obesity is accompanied by numerous adverse health effects, including cognitive decline and dementia (1, 2). Moreover, obesity has been linked with smaller brain tissue volumes, and decreased white matter integrity (2–5). On top of that, it has been found that obesity is associated with focal brain pathology in the form of cerebral small vessel disease (CSVD) (6).

An important limitation of previous studies is that obesity is generally assessed by body mass index (BMI) (2). However, aging is associated with a decrease in lean mass and an increase in fat mass, making BMI less suitable as an approximation of obesity in the elderly (2, 7). In a study looking at the association between body composition and metabolic syndrome the same “BMI problem” was encountered. Researchers found that fat mass index (FMI) was independently and positively associated with the presence of metabolic syndrome regardless of BMI and concluded that FMI is a better approximation of body composition (8). A second limitation is that previous studies mainly assessed cross-sectional relations, precluding inference on directionality.

We studied the association of body composition, divided into body mass, fat mass, and fat-free mass, with brain tissue volume, white matter integrity and markers of CSVD (white matter lesion hyperintensities (WMH), lacunes, and microbleeds) to evaluate whether fat mass and fat-free mass is a better approximation of body composition. Moreover, we aimed to assess the longitudinal association between body composition and brain health.

## Materials and Methods

### Setting

This study was embedded within the population-based Rotterdam Study. At study entry and subsequently every 3–4 years, all participants were invited to undergo extensive examinations. For the present study, 4,104 participants (2006–2014) underwent body composition assessment with a dual-energy X-ray absorptiometry (DXA) scan and a brain magnetic resonance imaging (MRI) scan (9). From this group we excluded participants with cortical infarcts on MRI (*N* = 164), cancer (*N* = 263), and dementia (*N* = 29), leaving 3,648 participants for the cross-sectional analyses. Between 2010 and 2017 in a subsequent examination-round, 1,844 participants had a follow-up MRI-scan available for the longitudinal analysis. Reasons of dropout at follow-up or unavailability of follow up MRI information have been included in Supplemental Figure 1.

The Rotterdam Study has received medical ethical approval according to the Population Screening Act: Rotterdam Study, executed by the Ministry of Health, Welfare, and Sports of the Netherlands. All participants provided written informed consent.

### Body Composition

Body weight and length were measured and BMI was calculated (kg/m^2^) (9). A DXA—scan (iDXA, GE Lunar Healthcare, USA) was performed to determine fat-free mass and fat mass in kilograms (9). From this, we calculated fat mass index (FMI) (kg/m^2^), and fat-free mass index (FFMI) (kg/m^2^).

### Brain Tissue Volume, White Matter Integrity, and Markers of CSVD

Brain-MRI was performed on a 1.5-tesla MRI scanner with a 8-channel head coil, using a standardized scan protocol which has been described in detail before (9, 10). To quantify brain tissue volume, gray matter volume, white matter volume, WMH volume, and intracranial volume, automated brain tissue classification was used. This quantification strategy was based on a *k*-nearest neighbor classifier algorithm, extended with an in-house-developed WMH segmentation (10). Total brain volume did not include the cerebellum (used MRI segmentation only segments supratentorial brain tissue and not all MRI scans incorporated the entire cerebellum in the field of view). T1-weighted MR images were processed using FreeSurfer (version 5.1) to obtain the hippocampus volume (9). White matter microstructure [fractional anisotropy (FA), mean diffusivity (MD)] was assessed with diffusion weighted imaging (9, 11). Visual ratings were performed for presence of lacunes or microbleeds (9).

### Covariables

Information on energy intake, smoking, alcohol, education, and physical activity was obtained from questionnaires and interviews as described in detail in the supplement (9).

### Statistics

We investigated associations of body composition with brain tissue volumes, WMH volume (log-transformed), and FA and MD as a ratio of intracranial volume (to adjust for absolute head size differences) using linear regression models. We calculated changes by subtracting baseline measurement from follow-up measurement, which were eventually standardized and used to assess the longitudinal association with linear regression models. The association of body composition with presence of lacunes and microbleeds at baseline and the progression of lacunes or microbleeds at follow-up for the longitudinal analysis was assessed with logistic regression models. Adjustments were made for age, age^2^, sex, and education (model 1). We additionally adjusted for energy intake, smoking, physical activity, and alcohol (model 2). Adding height, hypertension, hypercholesterolemia, and diabetes to the regression models did not change the effect estimates and were therefore left out of the final analysis. For the longitudinal analysis we additionally adjusted for time between MRI-scans. We ran the same analyses, with brain tissue volumes, WMH volume, and FA and MD unstandardized (while adjusting for intracranial volume in the regression model) to evaluate clinical relevance of the associations. We compared our results with previously reported brain tissue changes that ranged from 3.6 to 5.4 ml (these values represent estimated average changes over the head size distributions of the population studied) with 1 year of aging (12, 13).

We checked for interaction between body composition and sex and between body composition and age (<60 vs. ≥60 years) as effects of BMI might differ between mid- and late-life (7). We were powered (alpha level: 0.05; power level: 0.80) to detect minimal differences of 2.028 ml brain tissue volume. A *p*-value < 0.05 was considered statistically significant. IBM SPSS statistics version 24.0 (IBM Corp, Armonk, NY, USA) was used for analyses.

## Results

Baseline characteristics are described in Table 1. Mean age was 65.9 years (standard deviation (SD): 9.8), 57.3% was female. Mean BMI was 27.2 kg/m^2^ (SD: 3.9). Participants with a follow-up MRI scan compared to those with only baseline MRI were younger, higher educated, and healthier (Supplemental Table 1).

**Table 1:** Population characteristics.

**Characteristics**	**Population for cross-sectional analysis**	**Population for longitudinal analysis**
	**Baseline *N* = 3,648**	**Baseline *N* = 1,844**
Age, years	65.9 (11.1)	60.9 (9.9)
Age range, years	45.7–97.8	44.1–90.2
Female, %	57.3	55.9
Education level, %		
Primary	8.1	7.1
Lower	38.5	35.4
Middle	30.1	29.3
Higher	22.4	27.9
Hypertension, %	44.1	34.3
Hypercholesterolemia, %	50.1	46.9
Diabetes, %	8.4	7.4
Smoking, %		
Never	33.0	33.3
Former	52.1	49.7
Current	14.4	16.4
Physical activity, MET-hours per week	44.7 (18.0–83.8)^a^	46.3 (19.6–83.8)^a^
Energy intake, kcal/day	2,090.5 (1,689.6–2,571.6)^a^	2,2567.0 (1,777.8–2,647.2)^a^
Alcohol intake, g/day	7.3 (1.1–17.6)^a^	8.5 (1.7–20.0)^a^
**Body Composition**		
Length, cm	168.4 (9.4)	169.5 (9.4)
Weight, kg	77.4 (13.6)	77.9 (13.6)
Body mass index, kg/m^2^	27.2 (3.9)	27.0 (3.8)
Fat mass index, kg/m^2^	9.6 (3.3)	9.3 (3.3)
Fat free mass index, kg/m^2^	17.6 (2.1)	17.4 (2.2)
**Brain MRI Volumetry**		
Brain tissue volume, mL	930.2 (103.3)	949.4 (101.8)
Gray matter volume, mL	528.3 (60.8)	536.2 (61.9)
White matter volume, mL	401.8 (64.9)	413.2 (62.9)
Fractional anisotropy	0.3 (0.0)	0.3 (0.0)
Mean diffusivity	0.7 (0.0)	0.7 (0.0)
**Markers of Cerebral Small Vessel Disease**		
Lacunes, presence %	7.2	4.7
Microbleeds, presence %	19.7	16.1
White hyperintensity volume	8.2 (1.1)^*^	7.9 (1.0)^*^

*MET, metabolic equivalent of task; kcal, kilocalories; g, gram; cm, centimeter; kg, kilogram*;

m, meter; mL, milliliter. Values are mean (standard deviation) for continuous variables or median (interquartile range) when indicated (

a *), percentages for dichotomous variables*.

* *ln-transformed*.

We found that cross-sectionally a higher BMI and FMI were related with smaller white matter volumes (difference in Z-score per SD higher BMI: −0.064 [95% CI: −0.094, −0.035]; FMI: −0.067 [95% CI: −0.099, −0.034]), higher FA (BMI difference: 0.061 [95% CI: 0.031, 0.091]; FMI difference: 0.098 [95% CI: 0.065, 0.130]), and higher MD (BMI difference: 0.045 [95% CI: 0.018, 0.072]; FMI difference: 0.075 [95% CI: 0.045, 0.104]), and a higher FFMI was related with larger gray matter volumes (difference: 0.060 [95% CI: 0.018, 0.101]) (Table 2, model 1). Effect estimates did not change between the different models (Table 3, model 2), nor were there differences in effects of body composition indices between the various outcomes of interest. Though statistically significant, cross-sectional results might not be clinically relevant as effect estimates of change in brain tissue volumes, as well as their corresponding confidence intervals, reported in milliliter difference per one point increase in BMI, FMI, or FFMI are small and do not exceed clinically relevant amounts of tissue changes (Supplemental Tables 2, 3).

**Table 2:** The associations between body composition and brain tissue volume and CSVD markers—model 1.

	**Brain tissue volume**	**Gray matter volume**	**White matter volume**	**Hippocampus volume**	**Fractional anisotropy**	**Mean diffusivity**	White matter hyperintensity volume^*^	**Lacunes**	**Microbleeds**
	**Difference in Z-score**	**Difference in Z-score**	**Difference in Z-score**	**Difference in Z-score**	**Difference in Z-score**	**Difference in Z-score**	**Difference in Z-score**	**Odds ratio**	**Odds ratio**
	**(95% CI)**	**(95% CI)**	**(95% CI)**	**(95% CI)**	**(95% CI)**	**(95% CI)**	**(95% CI)**	**(95% CI)**	**(95% CI)**
	***p*****-value**	***p*****-value**	***p*****-value**	***p*****-value**	***p*****-value**	***p*****-value**	***p*****-value**	***p*****-value**	***p*****-value**
**CROSS-SECTIONAL**									
Body mass index (kg/m^2^)	−0.025	0.042	−0.064	−0.022	0.061	0.045	0.008	1.075	0.931
	(−0.050, 0.001)	(0.010, 0.073)	(−0.094, −0.035)	(−0.050, 0.006)	(0.031, 0.091)	(0.018, 0.072)	(−0.018, 0.034)	(0.944, 1.223)	(0.854, 1.015)
	0.056	0.009	0.000	0.128	0.000	0.001	0.565	0.276	0.103
Fat mass index (kg/m^2^)	−0.033	0.034	−0.067	−0.019	0.098	0.075	0.000	1.080	0.945
	(−0.061, −0.005)	(−0.001, 0.068)	(−0.099, −0.034)	(−0.015, 0.012)	(0.065, 0.130)	(0.045, 0.104)	(−0.028, 0.029)	(0.933, 1.250)	(0.859, 1.040)
	0.020	0.055	0.000	0.224	0.000	0.000	0.982	0.301	0.250
Fat free mass index (kg/m^2^)	−0.007	0.060	−0.059	−0.028	−0.022	−0.021	0.024	1.070	0.899
	(−0.040, 0.027)	(0.018, 0.101)	(−0.098, −0.021)	(−0.065, 0.0094)	(−0.062, 0.017)	(−0.056, 0.014)	(−0.010, 0.058)	(0.905, 1.264)	(0.804, 1.005)
	0.697	0.005	0.003	0.137	0.268	0.246	0.171	0.430	0.061
**LONGITUDINAL**									
Body mass index (kg/m^2^)	0.004	−0.006	0.011	−0.050	0.029	0.052	−0.004	1.003	0.978
	(−0.029, 0.036)	(−0.048, 0.036)	(−0.031, 0.054)	(−0.097, −0.004)	(−0.031, 0.089)	(−0.007, 0.111)	(−0.046, 0.038)	(0.816, 1.232)	(0.852, 1.123)
	0.810	0.789	0.604	0.034	0.339	0.084	0.861	0.979	0.754
Fat mass index (kg/m^2^)	0.012	0.004	0.011	−0.033	0.048	0.061	−0.009	0.906	0.921
	(−0.022, 0.047)	(−0.040, 0.049)	(−0.035, 0.056)	(−0.082, 0.017)	(−0.014, 0.110)	(0.000, 0.122)	(−0.054, 0.036)	(0.732, 1.120)	(0.800, 1.061)
	0.485	0.846	0.645	0.192	0.130	0.050	0.686	0.362	0.257
Fat free mass index (kg/m^2^)	−0.017	−0.031	0.012	−0.089	−0.026	0.017	0.010	1.149	1.080
	(−0.061, 0.027)	(−0.087, 0.026)	(−0.046, 0.069)	(−0.151, −0.026)	(−0.108, 0.055)	(−0.063, 0.098)	(−0.047, 0.067)	(0.946, 1.397)	(0.945, 1.233)
	0.448	0.291	0.684	0.006	0.527	0.672	0.726	0.162	0.259

* *ln-transformed. CI, confidence interval; kg, kilogram; m, meter. Cross-sectional differences represent the difference in standard deviation brain tissue volume (total brain tissue, gray matter, white matter, hippocampus volume, fractional anisotropy, mean diffusivity, and white matter hyperintensity volume) as a ratio of intracranial volume per standard deviation higher body mass index, fat mass index, or fat free mass index. Cross-sectional odds ratios represent the odds of the presence of lacunes and microbleeds per standard deviation higher body mass index, fat mass index, or fat free mass index. Longitudinal differences represent the difference in standard deviation change in brain tissue volume (total brain tissue, gray matter, white matter, hippocampus volume, fractional anisotropy, mean diffusivity, and white matter hyperintensity volume) as a ratio of intracranial volume per standard deviation higher body mass index, fat mass index, or fat free mass index. Longitudinal odds ratios represent the odds of progressing from no presence of lacunes and microbleeds to presence or the odds of progressing to more lacunes or microbleeds at follow-up per standard deviation higher body mass index, fat mass index, or fat free mass index. Effect estimates were considered statistically significant with a p-value < 0.05. Cross sectional models are adjusted for age, age^2^, sex, and education. Longitudinal models were additionally adjusted for time between MRI scans*.

**Table 3:** The associations between body composition and brain tissue volume and CSVD markers—model 2.

	**Brain tissue volume**	**Gray matter volume**	**White matter volume**	**Hippocampus volume**	**Fractional anisotropy**	**Mean diffusivity**	White matter hyperintensity volume^*^	**Lacunes**	**Microbleeds**
	**Difference in Z-score**	**Difference in Z-score**	**Difference in Z-score**	**Difference in Z-score**	**Difference in Z-score**	**Difference in Z-score**	**Difference in Z-score**	**Odds ratio**	**Odds ratio**
	**(95% CI)**	**(95% CI)**	**(95% CI)**	**(95% CI)**	**(95% CI)**	**(95% CI)**	**(95% CI)**	**(95% CI)**	**(95% CI)**
	***p*****-value**	***p*****-value**	***p*****-value**	***p*****-value**	***p*****-value**	***p*****-value**	***p*****-value**	***p*****-value**	***p*****-value**
**CROSS-SECTIONAL**									
Body mass index (kg/m^2^)	−0.028	0.039	−0.066	−0.016	0.078	0.060	0.013	1.134	0.918
	(−0.060, 0.003)	(0.001, 0.076)	(−0.101, −0.030)	(−0.049, 0.018)	(0.043, 0.112)	(0.029, 0.091)	(−0.018, 0.043)	(0.980, 1.131)	(0.830, 1.015)
	0.075	0.043	0.000	0.357	0.000	0.000	0.409	0.091	0.095
Fat mass index (kg/m^2^)	−0.035	0.033	−0.069	−0.008	0.114	0.092	0.006	1.137	0.927
	(−0.070, −0.001)	(−0.008, 0.075)	(−0.108, −0.030)	(−0.044, 0.029)	(0.076, 0.152)	(0.058, 0.127)	(−0.028, 0.039)	(0.962, 1.344)	(0.829, 1.038)
	0.045	0.113	0.001	0.685	0.000	0.000	0.728	0.131	0.188
Fat free mass index (kg/m^2^)	−0.014	0.051	−0.061	−0.034	−0.002	−0.010	0.028	1.149	0.895
	(−0.056, 0.027)	(0.002, 0.101)	(−0.107, −0.014)	(−0.077, 0.010)	(−0.047, 0.044)	(−0.051, 0.032)	(−0.012, 0.068)	(0.948, 1.392)	(0.786, 1.018)
	0.498	0.041	0.010	0.130	0.944	0.646	0.165	0.156	0.092
**LONGITUDINAL**									
Body mass index (kg/m^2^)	0.029	0.008	0.028	−0.041	0.004	0.025	0.005	0.943	0.979
	(−0.012, 0.069)	(−0.041, 0.057)	(−0.021, 0.077)	(−0.098, 0.016)	(−0.070, 0.078)	(−0.043, 0.092)	(−0.045, 0.055)	(0.725, 1.227)	(0.831, 1.154)
	0.165	0.756	0.270	0.163	0.918	0.475	0.840	0.662	0.804
Fat mass index (kg/m^2^)	0.039	0.005	0.032	−0.019	0.014	0.021	−0.004	0.835	0.948
	(−0.005, 0.083)	(−0.011, 0.021)	(−0.021, 0.085)	(−0.080, 0.042)	(−0.063, 0.091)	(−0.049, 0.091)	(−0.057, 0.0105)	(0.632, 1.104)	(0.801, 1.122)
	0.080	0.567	0.233	0.546	0.728	0.557	0.897	0.206	0.534
Fat free mass index (kg/m^2^)	0.003	−0.011	0.016	−0.093	−0.023	0.030	0.027	1.160	1.041
	(−0.052, 0.058)	(−0.078, 0.055)	(−0.051, 0.083)	(−0.171, −0.015)	(−0.126, 0.079)	(−0.063, 0.124)	(−0.041, 0.095)	(0.896, 1.501)	(0.885, 1.225)
	0.920	0.741	0.643	0.019	0.655	0.521	0.444	0.260	0.629

* *ln-transformed. CI, confidence interval; kg, kilogram; m, meter. Cross-sectional differences represent the difference in standard deviation brain tissue volume (total brain tissue, gray matter, white matter, and hippocampus volume) and the difference in standard deviation fractional anisotropy, mean diffusivity, and white matter hyperintensity volume as a ratio of intracranial volume per standard deviation higher body mass index, fat mass index, or fat free mass index. Cross-sectional odds ratios represent the odds of the presence of lacunes and microbleeds per standard deviation higher body mass index, fat mass index, or fat free mass index. Longitudinal differences represent the difference in standard deviation change in brain tissue volume (total brain tissue, gray matter, white matter, and hippocampus volume) and the difference in standard deviation fractional anisotropy, mean diffusivity, and white matter hyperintensity volume as a ratio of intracranial volume per standard deviation higher body mass index, fat mass index, or fat free mass index. Longitudinal odds ratios represent the odds of progressing from no presence of lacunes and microbleeds to presence or the odds of progressing to more lacunes or microbleeds at follow-up per standard deviation higher body mass index, fat mass index, or fat free mass index. Effect estimates were considered statistically significant with a p-value < 0.05. Cross sectional models are adjusted for age, age^2^, sex, education, energy intake, smoking, physical activity, and alcohol consumption. Longitudinal models were additionally adjusted for time between MRI scans*.

Only the longitudinal association between FFMI and change in hippocampus volume was statistically significant (difference: −0.089 [95% CI: −0.151, −0.026]) (Table 2; model 1), and did not change across models. However, it is questionable whether the direction of this association is biological plausible, and when reporting this association in milliliter (Supplemental Table 3; model 2), it is borderline statistically significant (difference in milliliter per one point increase in FFMI: −0.027 [95% CI: −0.055, 0.000]). We did not find any statistically significant or clinically relevant longitudinal associations between body composition, brain tissue volumes, white matter integrity, or markers of CSVD (Tables 2, 3; Supplemental Tables 2, 3). Both cross-sectional and longitudinal results did not differ between midlife and late life (data not shown), or between males and females (Supplemental Table 4).

## Discussion

We found that detailed measures of body composition (higher BMI and higher FMI) related to smaller white matter volume and decreased white matter microstructure and that higher FFMI related to higher gray matter volumes. However, expressing those cross-sectional associations in milliliter difference, raises the question whether those are also clinically relevant. No statistically significant or clinically relevant associations were found longitudinally (median follow-up: 5.5 years).

Strengths of this study are the large sample size, the distinction of body composition, the wide range of brain imaging markers, and the longitudinal design. However, some limitations should also be acknowledged. First, due to the observational design we cannot exclude the possibility of residual confounding. Second, we did not adjust for possible changes in body composition over time in the longitudinal analysis.

Previous studies showed that body composition relates to brain structure (14, 15), which is replicated in some of our cross-sectional results. Currently there are no established thresholds for rates of brain atrophy. However, in two other population-based studies, change in brain tissue volume with 1 year of aging were found to range from 3.6 to 5.4 ml (12, 13). Against this background, it is questionable whether a (cross-sectional) 0.496 ml smaller brain volume per one-point increase in BMI (independent of age) and other (longitudinal) effect estimates, are also clinically relevant. The absence of a longitudinal association did not result from insufficient power, as we were able to minimally detect a change of 2.028 ml brain tissue volume, which is smaller than a clinically relevant change of 5 ml brain volume (12). Though, the absence of a longitudinal association might result in part from selection bias. Indeed, it appeared that participants with MRI at follow-up compared to those without follow-up were younger, higher educated, and healthier. Another explanation might be that the effects of body composition in midlife and late life may be reversed (7, 16). But, we could not confirm effect modification by age in our study. Other longitudinal studies are inconclusive in their results, both reporting non-significant associations, and significant associations between body composition and various brain measurements (3, 5, 16–18), probably due to different methodologies. In a study investigating whether the effect of obesity on the brain increased with age they found no significant results and thus suggested that obesity may act as a modifier of brain atrophy rather than it being a direct cause (19). Cardiovascular risk factors (hypertension, glucose, cholesterol, plasma triglyceride) are known to co-occur frequently with obesity and have independent negative effects on brain structure (2, 20–22). Therefore, although we did not find significant associations between body composition and markers of CSVD, it might be that the effects of vascular risk factors may be less pronounced in their effects on brain structure. And it might also be that solely body composition does not influence brain health, but it is rather the interplay of cardiovascular risk factors altogether that may impact brain health negatively (23, 24).

Since BMI may not be a good approximation for body composition (25), we used FMI and FFMI. Surprisingly, both cross-sectionally and longitudinally, regression estimates between the different body composition measurement (BMI, FMI, and FFMI) and brain MRI data did not differ.

Based on our findings it seems that cross-sectional associations between body composition and brain health likely reflect cumulative risk or shared etiology. Moreover, it seems that body composition, decomposed into fat mass, and fat-free mass, does not influence brain changes or the presence of CSVD. Therefore, we cannot conclude that FMI and FFMI is a better approximation of adiposity than BMI to study brain health. Other longitudinal studies are needed to replicate our findings.

## Ethics Statement

The Rotterdam Study has been approved by the medical ethics committee according to the Population Screening Act: Rotterdam Study, executed by the Ministry of Health, Welfare, and Sports of the Netherlands. All participants provided written informed consent.

## Author Contributions

MI, FR, and MV: study design and funding. TV, MI, FR, DB, and MV: data collection. PC: data analysis. PC, TV, MI, DB, and MV: data interpretation. All authors: critical revision of the manuscript.

### Conflict of Interest Statement

The authors declare that the research was conducted in the absence of any commercial or financial relationships that could be construed as a potential conflict of interest.
