# Supplementary material for: Body Composition Is Not Related to Structural or Vascular Brain Changes
Source: Front Neurol. 2019 May 28;10:559. doi: 10.3389/fneur.2019.00559 (PMC6546802; doi:10.3389/fneur.2019.00559)
Supplement: Supplementary file 1 [file Data_Sheet_1.pdf]

Supplemental tables

Supplemental table 1. Baseline characteristics of participants with and without follow-up MRI scan

Characteristics	Only baseline MRI available	FU MRI available
	Baseline N = 1,804	Baseline N = 1,844
Age, years	71.0 (9.8)†	60.9 (9.9) †
Female, %	58.8	55.8
Education level, %		
Primary	9.2	7.1
Lower	42.3	35.5
Middle	31.3	29.4
Higher	17.0†	28.0†
Hypertension, %	54.2†	34.3†
Hypercholesterolemia, %	53.9†	47.4†
Diabetes, %	9.5†	7.5†
Smoking, %		
Never	32.9	33.5
Former	54.7	50.0
Current	12.4†	16.5†
Physical activity, MET-hours per week	42.8 (17.5 – 83.8)[a]	46.3 (19.6 – 83.8)[a]
Energy intake, kcal/day	2021.5 (1586.8 – 2468.6)[a]†	2161.5 (1777.8 – 2647.2)[a]†
Alcohol intake, g/d	10.9 (0.6 – 16.1)[a]†	8.5 (1.7 – 20.0)[a]†

Values are mean (standard deviation) for continuous variables or median (interquartile range) when indicated ([a]), percentages for dichotomous variables. T-test were used for normally distributed variables, χ^2-test for dichotomous variables, and Mann-Whitney U-Test for non-normally distributed variables to see whether characteristics were significantly different ($p < 0.05$) between participants with only a baseline MRI-scan and participants with a second MRI-scan at follow-up.

† Statistically significant difference ($p < 0.05$) between the groups.

Supplemental table 2. The associations between body composition and brain tissue volume – model 1

	**Brain tissue volume**	**Gray matter volume**	**White matter volume**	**Hippocampus volume**	**Fractional anisotropy**	**Mean diffusivity**	**White matter hyperintensity volume***
	Difference in mL<br>(95% CI)<br>p-value	Difference in mL<br>(95% CI)<br>p-value	Difference in mL<br>(95% CI)<br>p-value	Difference in mL<br>(95% CI)<br>p-value	Difference<br>(95% CI)<br>p-value	Difference<br>(95% CI)<br>p-value	Difference<br>(95% CI)<br>p-value
				Cross-sectional			
Body mass index (kg/m^2)	-0.429<br>(-0.767, -0.091)<br>0.013	0.297<br>(-0.028, 0.622)<br>0.073	-0.726<br>(-1.088, -0.364)<br>0.000	-0.010<br>(-0.017, -0.003)<br>0.003	0.000<br>(0.000, 0.000)<br>0.296	0.000<br>(0.000, 0.000)<br>0.242	0.005<br>(-0.001, 0.012)<br>0.121
Fat mass index (kg/m^2)	-0.784<br>(-1.233, -0.335)<br>0.001	0.140<br>(-0.292, 0.573)<br>0.525	-0.925<br>(-1.406, -0.443)<br>0.000	-0.016<br>(-0.025, -0.007)<br>0.001	0.000<br>(0.000, 0.000)<br>0.095	0.000<br>(0.000, 0.000)<br>0.483	0.006<br>(-0.003, 0.015)<br>0.203
Fat free mass index (kg/m^2)	0.083<br>(-0.743, 0.910)<br>0.844	1.302<br>(0.508, 2.095)<br>0.001	-1.219<br>(-2.105, -0.332)<br>0.007	-0.007<br>(-0.024, 0.009)<br>0.384	0.000<br>(0.000, 0.000)<br>0.601	0.000<br>(-0.001, 0.000)<br>0.118	0.013<br>(-0.004, 0.030)<br>0.147
				Longitudinal			
Body mass index (kg/m^2)	-0.064<br>(-0.435, 0.307)<br>0.735	-0.084<br>(-0.474, 0.306)<br>0.674	0.020<br>(-0.347, 0.387)<br>0.916	-0.010<br>(-.019, -0.001)<br>0.038	0.000<br>(0.000, 0.000)<br>0.997	0.000<br>(0.000, 0.000)<br>0.662	0.008<br>(-0.014, 0.029)<br>0.479
Fat mass index (kg/m^2)	-0.080<br>(-0.559, 0.400)<br>0.745	-0.044<br>(-0.547, 0.460)<br>0.865	-0.036<br>(-0.510, 0.437)<br>0.881	-0.009<br>(-0.020, 0.003)<br>0.143	0.000<br>(0.000, 0.000)<br>0.859	0.000<br>(0.000, 0.000)<br>0.409	0.003<br>(-0.024, 0.030)<br>0.832
Fat free mass index (kg/m^2)	-0.102<br>(-1.035, 0.831)<br>0.830	-0.364<br>(-1.344, 0.616)<br>0.467	0.262<br>(-0.660, 1.183)<br>0.578	-0.027<br>(-0.050, -0.004)<br>0.019	0.000<br>(0.000, 0.000)<br>0.710	0.000<br>(0.000, 0.001)<br>0.575	0.037<br>(-0.016, 0.090)<br>0.171

* ln-transformed. CI: confidence interval. kg: kilogram. m: meter. mL: milliliter. Cross-sectional differences represent the difference in milliliter brain tissue volume (total brain tissue, gray matter, white matter, and hippocampus volume) and the difference in fractional anisotropy, mean diffusivity and white matter hyperintensity volume per one point increase in body mass index, fat mass index, or fat free mass index. Longitudinal differences

represent the difference in milliliter change in brain tissue volume (total brain tissue, gray matter, white matter, and hippocampus volume) and the difference in fractional anisotropy, mean diffusivity and white matter hyperintensity volume per one point increase in body mass index, fat mass index, or fat free mass index. Effect estimates were considered statistically significant with a p-value < 0.05. Cross sectional models are adjusted for age, age^2, intracranial volume, sex, and education. Longitudinal models were additionally adjusted for time between MRI scans.

Supplemental table 3. The associations between body composition and brain tissue volume – model 2

	Brain tissue volume	Gray matter volume	White matter volume	Hippocampus volume	Fractional anisotropy	Mean diffusivity	White matter hyperintensity volume*
	Difference in mL (95% CI) p-value	Difference in mL (95% CI) p-value	Difference in mL (95% CI) p-value	Difference in mL (95% CI) p-value	Difference (95% CI) p-value	Difference (95% CI) p-value	Difference (95% CI) p-value
				Cross-sectional			
Body mass index (kg/m^2)	-0.496 (-0.913, -0.079) 0.020	0.247 (-0.145, 0.638) 0.217	-0.743 (-1.179, -0.306) 0.001	-0.010 (-0.018, -0.002) 0.019	0.000 (0.000, 0.000) 0.281	0.000 (0.000, 0.000) 0.261	0.007 (-0.001, 0.015) 0.096
Fat mass index (kg/m^2)	-0.843 (-1.401, -0.284) 0.003	0.124 (-0.400, 0.648) 0.642	-0.967 (-1.551, -0.383) 0.001	-0.013 (-0.024, -0.003) 0.014	0.000 (0.000, 0.000) 0.128	0.000 (0.000, 0.000) 0.694	0.008 (-0.003, 0.019) 0.159
Fat free mass index (kg/m^2)	-0.153 (-1.175, 0.868) 0.768	1.061 (0.104, 2.018) 0.030	-1.214 (-2.283, -0.145) 0.026	-0.012 (-0.032, 0.007) 0.212	0.000 (0.000, 0.000) 0.872	-0.001 (-0.001, 0.000) 0.043	0.015 (-0.005, 0.035) 0.135
				Longitudinal			
Body mass index (kg/m^2)	0.296 (-0.165, 0.758) 0.208	0.086 (-0.375, 0.548) 0.713	0.210 (-0.218, 0.638) 0.336	-0.007 (-0.018, 0.004) 0.218	0.000 (0.000, 0.000) 0.779	0.000 (0.000, 0.000) 0.373	0.008 (-0.017, 0.033) 0.531
Fat mass index (kg/m^2)	0.394 (-0.205, 0.994) 0.197	0.139 (-0.461, 0.738) 0.650	0.256 (-0.300, 0.812) 0.367	-0.004 (-0.019, 0.010) 0.561	0.000 (0.000, 0.000) 0.785	0.000 (0.000, 0.000) 0.172	0.006 (-0.027, 0.038) 0.726
Fat free mass index (kg/m^2)	0.398 (-0.769, 1.565) 0.503	0.027 (-1.140, 1.193) 0.964	0.372 (-0.710, 1.453) 0.501	-0.028 (-0.056, 0.000) 0.049	0.000 (0.000, 0.000) 0.869	0.000 (0.000, 0.001) 0.618	0.028 (-0.034, 0.091) 0.374

* ln-transformed. CI: confidence interval. kg: kilogram. m: meter. mL: milliliter. Cross-sectional differences represent the difference in milliliter brain tissue volume (total brain tissue, gray matter, white matter, and hippocampus volume) and the difference in fractional anisotropy, mean diffusivity and white matter hyperintensity volume per one point increase in body mass index, fat mass index, or fat free mass index. Longitudinal differences represent the

difference in milliliter change in brain tissue volume (total brain tissue, gray matter, white matter, and hippocampus volume) and the difference in fractional anisotropy, mean diffusivity and white matter hyperintensity volume per one point increase in body mass index, fat mass index, or fat free mass index. Effect estimates were considered statistically significant with a p-value < 0.05. Cross sectional models are adjusted for age, age^2, intracranial volume, sex, education, energy intake, smoking, physical activity, and alcohol consumption. Longitudinal models were additionally adjusted for time between MRI scans.

Supplemental table 4. The associations between body composition and brain tissue volume and CSVD markers per sex

	**Brain tissue volume**	**Gray matter volume**	**White matter volume**	**Hippocampus volume**	**Fractional anisotropy**	**Mean diffusivity**	**White matter hyperintensity volume***	**Lacunes**	**Microbleeds**
	Difference in Z-score (95% CI) p-value	Difference in Z-score (95% CI) p-value	Difference in Z-score (95% CI) p-value	Difference in Z-score (95% CI) p-value	Difference in Z-score (95% CI) p-value	Difference in Z-score (95% CI) p-value	Difference in Z-score (95% CI) p-value	Odds ratio (95% CI) p-value	Odds ratio (95% CI) p-value
					Cross-sectional				
Males (N = 1,557)									
Body mass index (kg/m^2)	-0.055<br>(-0.111, 0.002)<br>0.059	0.047<br>(-0.027, 0.122)<br>0.212	-0.102<br>(-0.176, -0.029)<br>0.006	0.023<br>(-0.052, 0.078)<br>0.687	0.028<br>(-0.037, 0.092)<br>0.397	0.017<br>(-0.040, 0.075)<br>0.550	-0.021<br>(-0.080, 0.039)<br>0.494	1.196<br>(0.903, 1.583)<br>0.213	0.852<br>(0.704, 1.032)<br>0.101
Fat mass index (kg/m^2)	-0.092<br>(-0.155, -0.028)<br>0.005	0.025<br>(-0.059, 0.109)<br>0.561	-0.124<br>(-0.207, -0.042)<br>0.003	0.010<br>(-0.064, 0.083)<br>0.794	0.079<br>(0.006, 0.151)<br>0.033	0.067<br>(0.003, 0.131)<br>0.041	-0.009<br>(-0.076, 0.058)<br>0.799	1.139<br>(0.823, 1.578)<br>0.433	0.849<br>(0.684, 1.054)<br>0.138
Fat free mass index (kg/m^2)	0.011<br>(-0.051, 0.072)<br>0.735	0.069<br>(-0.012, 0.150)<br>0.097	-0.048<br>(-0.128, 0.032)<br>0.240	0.015<br>(-0.055, 0.086)<br>0.669	-0.053<br>(-0.123, 0.018)<br>0.141	-0.059<br>(-0.121, 0.004)<br>0.065	-0.033<br>(-0.098, 0.031)<br>0.314	1.246<br>(0.912, 1.703)<br>0.167	0.888<br>(0.724, 1.090)<br>0.257
Females (N = 2,091)									
Body mass index (kg/m^2)	-0.001<br>(-0.042, 0.040)<br>0.964	0.041<br>(-0.005, 0.087)<br>0.083	-0.037<br>(-0.079, 0.005)<br>0.087	-0.024<br>(-0.066, 0.017)<br>0.251	0.090<br>(0.046, 0.134)<br>0.000	0.040<br>(0.000, 0.080)<br>0.048	0.005<br>(-0.033, 0.042)<br>0.813	1.045<br>(0.862, 1.267)<br>0.651	0.909<br>(0.800, 1.033)<br>0.143
Fat mass index (kg/m^2)	0.005<br>(-0.040, 0.049)<br>0.844	0.045<br>(-0.005, 0.095)<br>0.080	-0.034<br>(-0.080, 0.012)<br>0.144	-0.013<br>(-0.059, 0.032)<br>0.568	0.124<br>(0.077, 0.172)<br>0.000	0.068<br>(0.025, 0.111)<br>0.002	-0.009<br>(-0.050, 0.032)<br>0.681	1.031<br>(0.830, 1.281)<br>0.782	0.909<br>(0.789, 1.046)<br>0.183
Fat free mass index (kg/m^2)	-0.017<br>(-0.078, 0.044)<br>0.590	0.039<br>(-0.030, 0.108)<br>0.267	-0.053<br>(-0.115, 0.010)<br>0.098	-0.062<br>(-0.124, 0.000)<br>0.048	0.010<br>(-0.056, 0.075)<br>0.768	-0.032<br>(-0.091, 0.027)<br>0.289	0.043<br>(-0.013, 0.099)<br>0.128	1.100<br>(0.832, 1.453)<br>0.503	0.886<br>(0.736, 1.067)<br>0.201

	Longitudinal								
Males (N = 814)									
Body mass index (kg/m^2)	0.044<br>(-0.066, 0.154)<br>0.436	0.032<br>(-0.077, 0.142)<br>0.564	0.020<br>(-0.079, 0.118)<br>0.696	0.000<br>(-0.107, 0.108)<br>0.997	0.047<br>(-0.085, 0.180)<br>0.485	0.027<br>(-0.091, 0.146)<br>0.648	-0.011<br>(-0.094, 0.071)<br>0.786	0.824<br>(0.503, 1.348)<br>0.440	0.951<br>(0.699, 1.294)<br>0.750
Fat mass index (kg/m^2)	0.088<br>(-0.034, 0.210)<br>0.156	0.094<br>(-0.028, 0.215)<br>0.130	0.008<br>(-0.101, 0.118)<br>0.879	0.013<br>(-0.106, 0.131)<br>0.832	0.082<br>(-0.058, 0.222)<br>0.252	0.095<br>(-0.029, 0.220)<br>0.133	-0.002<br>(-0.094, 0.090)<br>0.959	0.847<br>(0.495, 1.450)<br>0.545	0.784<br>(0.550, 1.118)<br>0.179
Fat free mass index (kg/m^2)	-0.035<br>(-0.156, 0.086)<br>0.569	-0.069<br>(-0.019, 0.051)<br>0.259	0.032<br>(-0.077, 0.140)<br>0.569	-0.019<br>(-0.137, 0.099)<br>0.750	-0.030<br>(-0.175, 0.115)<br>0.685	-0.096<br>(-0.225, 0.033)<br>0.143	-0.022<br>(-0.114, 0.069)<br>0.632	0.831<br>(0.484, 1.427)<br>0.502	1.279<br>(0.909, 1.800)<br>0.158
Females (N = 1,030)									
Body mass index (kg/m^2)	0.014<br>(-0.012, 0.040)<br>0.287	-0.024<br>(-0.078, 0.029)<br>0.368	0.044<br>(-0.016, 0.104)<br>0.146	-0.038<br>(-0.112, 0.036)<br>0.314	0.011<br>(-0.088, 0.111)<br>0.826	0.023<br>(-0.069, 0.115)<br>0.622	-0.054<br>(-0.122, 0.015)<br>0.124	0.889<br>(0.627, 1.262)<br>0.512	0.912<br>(0.737, 1.127)<br>0.393
Fat mass index (kg/m^2)	0.013<br>(-0.014, 0.041)<br>0.344	-0.024<br>(-0.080, 0.032)<br>0.400	0.043<br>(-0.020, 0.106)<br>0.183	-0.010<br>(-0.088, 0.068)<br>0.794	0.012<br>(-0.090, 0.115)<br>0.812	-0.006<br>(-0.101, 0.089)<br>0.900	-0.057<br>(-0.129, 0.015)<br>0.123	0.762<br>(0.512, 1.135)<br>0.181	0.919<br>(0.734, 1.152)<br>0.465
Fat free mass index (kg/m^2)	0.019<br>(-0.022, 0.060)<br>0.356	-0.029<br>(-0.112, 0.054)<br>0.492	0.056<br>(-0.038, 0.149)<br>0.242	-0.135<br>(-0.250, -0.021)<br>0.021	0.006<br>(-0.145, 0.158)<br>0.935	0.120<br>(-0.020, 0.259)<br>0.092	-0.051<br>(-0.157, 0.056)<br>0.353	1.307<br>(0.786, 2.174)<br>0.302	0.871<br>(0.627, 1.208)<br>0.407

* ln-transformed. CI: confidence interval. kg: kilogram. m: meter. Cross-sectional differences represent the difference in standard deviation brain tissue volume (total brain tissue, gray matter, white matter, and hippocampus volume) and the difference in standard deviation fractional anisotropy, mean diffusivity and white matter hyperintensity volume as a ratio of intracranial volume per standard deviation higher body mass index, fat mass index, or fat free mass index. Cross-sectional odds ratios represent the odds of the presence of lacunes and microbleeds per standard deviation higher body mass index, fat mass index, or fat free mass index. Longitudinal differences represent the difference in standard deviation change in brain tissue volume (total brain tissue, gray matter, white matter, and hippocampus volume,) and the difference in standard deviation fractional anisotropy, mean diffusivity and white matter hyperintensity volume as a ratio of intracranial volume per standard deviation higher body mass index, fat mass index, or fat free mass index. Longitudinal odds ratios represent the odds of progressing from no presence of lacunes and microbleeds to presence or the odds of progressing to more lacunes or microbleeds at follow-up per standard deviation higher body mass index, fat mass index, or fat free mass index. Effect estimates were considered statistically significant with a p-value < 0.05. Cross sectional models are adjusted for age, age^2, sex, education, energy intake, smoking, physical activity, alcohol intake, diabetes, hypertension and hypercholesterolemia. Longitudinal models were additionally adjusted for time between MRI scans.

Supplemental figure 1. Dropout reasons of the Rotterdam Study and completeness of MRI data at follow-up

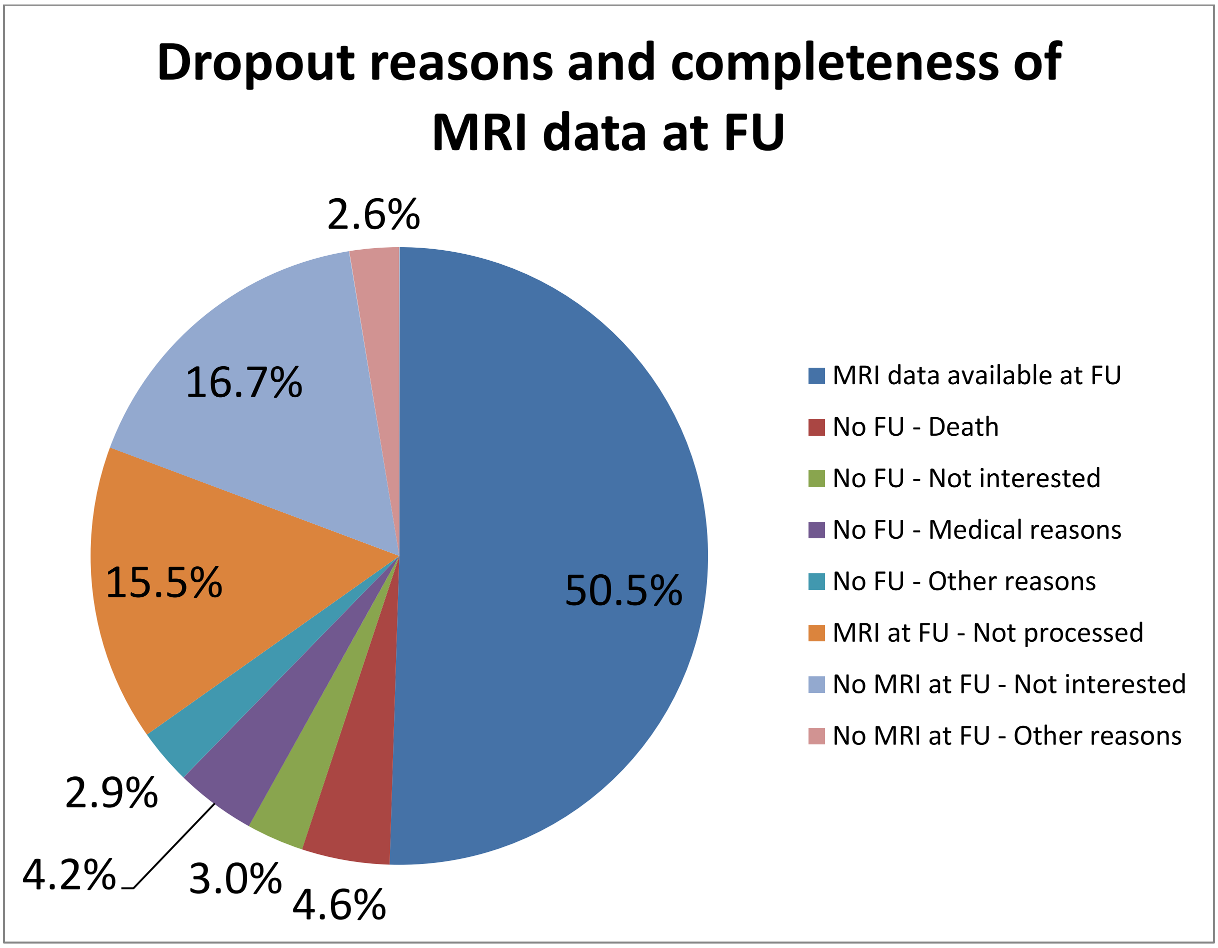

FU: follow-up. MRI: magnetic resonance imaging. At baseline there were 3,648 participants with information on body composition and brain measurements of which 1,844 (50.5%) had MRI data available at follow-up, and 1,804 participants had no MRI data available at follow-up. Of those 1,804 participants, 166 (4.6%) participants died during follow-up, 110 (3.0%) were not interested in participating in the Rotterdam Study again, 152 (4.2%) participants did not participate any longer in the Rotterdam Study due to medical reasons, and 108 (2.9%) participants did not want to participate in the Rotterdam Study any longer due to other reasons. A part of those 1,804 participants did have a follow-up research visit but did not have MRI data available at follow-up (N = 1,268). Of those 1,268 participants, 564 (15.5%) participants did underwent follow-up MRI scanning of the brain, but their scans are not processed, 610 (16.7%) participants were specifically not interested in participating in the follow-up MRI scan, and 94 (2.6%) participants had other reasons (medical condition, contraindication, afraid of going into the MRI scanner) to not participate in the follow-up MRI scan.

Supplemental Material and Methods

Covariables

Covariables were chosen based on the criteria for a confounding factor: 1) a confounding factor must be a risk factor for the outcome under study; 2) a confounding factor must be associated with the exposure under study in the source population; 3) a confounding factor must not be affected by the exposure or the outcome. In particular, it cannot be an intermediate factor in the causal pathway between the exposure and the outcome (Rothman et al., 2008). Initially analyses were run with the following factors in the model: age, sex, education, energy intake, smoking, alcohol intake, physical activity, prevalent diabetes, hypertension, and hypercholesterolemia. As diabetes, hypertension, and hypercholesterolemia may be an intermediate factor on the causal pathway between body composition and brain health, and as adding those factors to the regression models did not change effect estimates (see also main manuscript section Results), we have left those out of the final model to limit the possibilities of (erroneous) over adjustment. However, the other factors (energy intake, smoking, alcohol intake, and physical activity) besides the fixed demographic variables (age, sex, education) are known to be both associated with the exposure (body composition) and the outcome (brain health) and thus we have added them as confounding factors in our regression models (Khosla and Lowe, 1971; Romieu et al., 1988; Crews, 1999; Crews et al., 2004; Titova et al., 2013; Traversy and Chaput, 2015; Chin et al., 2016; Tan et al., 2017; Croll et al., 2018).

Educational level was categorized as primary, lower, middle, or higher (Ikram et al., 2017). Dietary intake was assessed with a validated, self-administered, semiquantitative food-frequency questionnaire (FFQ) consisting of 389 items. For different food items, questions about the number of servings per day and the frequency of consumption were included. Energy intake was calculated using the Dutch Food Composition Table (NEVO) (Voortman et al., 2017). Based on the information obtained from the FFQ, alcohol intake in grams was calculated. Information on smoking was collected through self-report and categorized into never, former, and current smoking (Ikram et al., 2017). The LASA (Longitudinal Aging Study Amsterdam) Physical Activity Questionnaire was used to assess the amount of physical activity. For each participant, data were recalculated into MET (metabolic equivalent of task) hours per week (Ainsworth et al., 2011).

Supplemental references

Ainsworth, B.E., Haskell, W.L., Herrmann, S.D., Meckes, N., Bassett, D.R., Jr., Tudor-Locke, C., et al. (2011). 2011 Compendium of Physical Activities: a second update of codes and MET values. *Med Sci Sports Exerc* 43(8)**,** 1575-1581.

Chin, S.H., Kahathuduwa, C.N., and Binks, M. (2016). Physical activity and obesity: what we know and what we need to know. *Obes Rev* 17(12)**,** 1226-1244.

Crews, F.T. (1999). Alcohol and neurodegeneration. *CNS Drug Reviews* 5(4)**,** 379-394.

Crews, F.T., Collins, M.A., Dlugos, C., Littleton, J., Wilkins, L., Neafsey, E.J., et al. (2004). Alcohol-induced neurodegeneration: when, where and why? *Alcoholism: Clinical and Experimental Research* 28(2)**,** 350-364.

Croll, P.H., Voortman, T., Ikram, M.A., Franco, O.H., Schoufour, J.D., Bos, D., et al. (2018). Better diet quality relates to larger brain tissue volumes: The Rotterdam Study. *Neurology* 90(24)**,** e2166-e2173.

Ikram, M.A., Brusselle, G.G.O., Murad, S.D., van Duijn, C.M., Franco, O.H., Goedegebure, A., et al. (2017). The Rotterdam Study: 2018 update on objectives, design and main results. *Eur J Epidemiol*.

Khosla, T., and Lowe, C.R. (1971). Obesity and smoking habits. *Br Med J* 4(5778)**,** 10-13.

Romieu, I., Willett, W.C., Stampfer, M.J., Colditz, G.A., Sampson, L., Rosner, B., et al. (1988). Energy intake and other determinants of relative weight. *The American journal of clinical nutrition* 47(3)**,** 406-412.

Rothman, K.J., Greenland, S., and Lash, T.L. (2008). *Modern epidemiology*. Wolters Kluwer Health/Lippincott Williams & Wilkins Philadelphia.

Tan, Z.S., Spartano, N.L., Beiser, A.S., DeCarli, C., Auerbach, S.H., Vasan, R.S., et al. (2017). Physical Activity, Brain Volume, and Dementia Risk: The Framingham Study. *J Gerontol A Biol Sci Med Sci* 72(6)**,** 789-795.

Titova, O.E., Ax, E., Brooks, S.J., Sjögren, P., Cederholm, T., Kilander, L., et al. (2013). Mediterranean diet habits in older individuals: Associations with cognitive functioning and brain volumes. *Experimental Gerontology* 48(12)**,** 1443-1448. doi: https://doi.org/10.1016/j.exger.2013.10.002.

Traversy, G., and Chaput, J.P. (2015). Alcohol Consumption and Obesity: An Update. *Curr Obes Rep* 4(1)**,** 122-130.

Voortman, T., Kiefte-de Jong, J.C., Ikram, M.A., Stricker, B.H., van Rooij, F.J.A., Lahousse, L., et al. (2017). Adherence to the 2015 Dutch dietary guidelines and risk of non-communicable diseases and mortality in the Rotterdam Study. *European Journal of Epidemiology*. doi: 10.1007/s10654-017-0295-2.
